# Acute beneficial effects of a functional energy shot on cognitive performance and mood states during cognitively demanding task performance: a randomized, double-blind, placebo-controlled, crossover trial

**DOI:** 10.3389/fnut.2024.1496092

**Published:** 2025-01-09

**Authors:** Olivia K. O’Shea, Nathan Lawley, Anna Azzopardi, Allison Gutkowski, Michelle Murphy Niedziela, Rachel Horn, David O. Kennedy, Jason Adamson

**Affiliations:** ^1^Research and Development, The Water Street Collective, London, United Kingdom; ^2^Research and Development, British American Tobacco (Investments) Ltd., Southampton, United Kingdom; ^3^HCD Research, Flemington, NJ, United States; ^4^Brain, Performance, Nutrition Research Centre, Northumbria University, Newcastle upon Tyne, United Kingdom

**Keywords:** energy, mood, cognition, performance, trial, supplement

## Abstract

**Introduction:**

Functional beverages are increasingly popular but it is important to validate their purported effects through research. The aim of the current study was to investigate the effects of a new functional energy shot on cognitive performance and mood states in healthy adults, with a focus on measuring mental energy enhancement and attenuation of negative effects associated with extended performance of mentally demanding tasks.

**Methods:**

This study was a randomized, double-blind, placebo-controlled, crossover trial. Thirty-seven healthy males and females, aged 18–30 years, consumed a functional energy shot (Ryde: Energize) or placebo on separate testing occasions one-week apart. Participants completed cognitive and mood assessments pre-dose, and then 30 minutes post-dose over the course of approximately 2 hours. The functional energy shot contained caffeine, ginseng, vitamins and taurine, while the placebo shot was matched for flavor but did not contain these additional ingredients.

**Results:**

Use of the functional energy shot was associated with significant improvements over placebo in cognitive performance, as measured by the Cognitive Demand Battery, with enhanced global performance, task-specific accuracy and speed across repeated assessments. Additionally, the shot mitigated negative effects associated with extended cognitive tasks, reducing perceived mental fatigue and increasing perceived alertness and energy. Working memory tasks showed faster performance post-consumption, and mood assessments revealed positive effects on vigor, fatigue and overall mood disturbance.

**Discussion:**

These results indicate wide cognitive and mood effects of this functional energy shot, potentially attributable to synergistic combination of active ingredients.

**Clinical trial registration:**

clinicaltrials.gov, identifier NCT06384586.

## Introduction

Functional beverages and shots which can offer additional benefits beyond basic hydration and nutritional needs, are becoming increasingly popular with a wide range of consumers who are looking for convenient portable formats that deliver putative benefits such as increased focus, relaxation or energy (1). However, previous research evaluating the functional benefits of consuming energy drinks has yielded varied outcomes. While some research indicates enhancements in aspects of mood and cognitive performance (2–4), other studies have shown no notable effects (5). These differences likely stem from the different formulations of these products as well as varying protocols and outcome measures used in these studies. This highlights the importance of testing products for efficacy using rigorous study designs. Despite this, the vast majority of these types of products have not been subject to clinical research to confirm their proposed effects.

A number of bioactive ingredients are commonly incorporated into functional beverages. The subject ingredients here are caffeine, Panax ginseng extract, water-soluble vitamins and taurine. Caffeine is the most widely consumed psychoactive substance globally, and is found in many natural plant products, such as tea, coffee, and cocoa. It is commonly added to functional products both for its psychoactive properties and its ability to reinforce taste and sensorial preferences of the drink or food vehicles it is consumed in. Caffeine’s psychoactive properties are generally attributed to its inhibition of the general inhibitory neuromodulator adenosine by occupying adenosine’s A_1_ and A_2*A*_ receptors in the brain. This results in a net increase in activity associated with a number of neurotransmitter systems. In behavioral terms, caffeine, when taken alone, has relatively consistent improvements in mental and physical performance. However, these are generally restricted to increased subjective alertness/arousal and, in terms of mental performance, consistent improvements in the performance of tasks assessing reaction time or focused attention/vigilance (6–8).

Extracts of members of the Panax genus, commonly referred to as ginseng, are also common functional beverage ingredients. The active components, triterpene “ginsenosides,” have been shown to interact with mammalian hormone receptors due to the structural similarities between ginsenosides and mammalian hormones (9). In particular, ginsenosides interact with stress-regulating glucocorticoid receptors, which are present in nearly every cell of the body, including in the brain. Downstream effects of these interactions include upregulation of the synthesis of the signaling molecule nitric oxide and indirect modulation of the activity associated with numerous neurotransmitters, including GABA, serotonin, glutamate, and acetylcholine (10, 11). Single doses of standardized ginseng extracts have been shown to have consistent performance enhancing effects on brain function (10), including during extended performance of mentally demanding tasks (12, 13).

One further potential route toward enhancing brain function is supplementation with water-soluble vitamins (i.e., B vitamins and vitamin C). These essential micronutrients contribute substantially to cellular metabolic functioning as co-factors for enzymes that drive the functioning of the cellular cycles that generate energy and synthesize bioactive molecules (14). B vitamins have notable effects in the brain, which is one of the most metabolically active organs in the body. Here they are essential for the synthesis of many neurochemicals including neuromodulators and regulators of cerebral blood-flow such as nitric oxide (15). Water-soluble vitamins have also been shown to modulate human brain function after a single dose and to engender mood and cognitive benefits following longer-term supplementation (15–17).

Finally, taurine is a non-proteinogenic amino acid that plays ubiquitous roles in metabolism. The body’s natural synthesis of this compound can also be augmented by dietary supplementation. Whilst the benefits of taurine supplementation to brain function are currently unclear, a meta-analysis of the ergogenic properties of caffeine and taurine containing energy drinks concluded that their physical performance effects were positively related to the amount of taurine in the drink, rather than the caffeine content. This suggests that taurine could play a contributory role in the ergogenic efficacy of energy drinks (18).

One final factor that should be considered is that caffeine, due to its structural similarity to adenosine, which is itself a ubiquitous mediator and factor in cellular function, also enjoys potential interactive properties with a wide range of other bioactive compounds. This raises the possibility that combining caffeine with other bioactive compounds, including those noted above, may lead to interactive or synergistic effects that exceed those associated with caffeine alone (19).

Previous research has suggested that one particularly sensitive paradigm for assessing the brain function effects of nutritional interventions, and one which mirrors many real-world demands, is the extended performance of cognitively demanding tasks. Performance of demanding tasks, by their nature, may be rate-limited by physical brain resources. As an example of one such paradigm, the Cognitive Demand Battery (CDB) employed in the current study is composed of tasks that correspond closely with the task requirements for measuring the construct of “mental energy” (20) and the battery’s application has been shown to engender consistent negative effects, most often in terms of mental fatigue. The CDB has also been shown to be sensitive to the psychopharmacological effects of multiple phytochemicals, including ginseng extracts (12, 13), water-soluble vitamins (21), and products combining caffeine with other bioactive compounds (19, 22). Therefore, this study was conducted in order to assess whether a functional energy shot containing caffeine, taurine, ginseng, and water-soluble vitamins could enhance task performance and attenuate the negative consequences of extended (60 min) performance of cognitively demanding tasks.

## Materials and methods

### Design

This was a randomized, double-blind, placebo-controlled, two-arm, crossover study investigating the acute effects of a 60 ml functional energy shot on cognitive and mood states measures reflecting mental energy. There was a 7-day wash-out period between the counterbalanced consumption of the two study products (a functional energy shot and a flavor-matched placebo shot).

### Participants

The study was conducted in Iselin, NJ, United States. Forty-five healthy participants (recruited via an existing study recruitment database and advertisements) who were habitual caffeine consumers (200–500 mg daily) were enrolled in the study [16 females, 29 males; mean age: 24.29 (±3.44) years (range 18–30 years)]. Exclusion criteria included self-reported sleep disturbances, gastrointestinal issues, irregular heartbeats or other heart conditions, moderate or severe anxiety or depression, color-blindness, dyscalculia or paralysis of the upper body, the use recreational or non-prescription drugs or painkillers or medication other than contraceptives. Participants who used green tea extract, diet medications, appetite suppressors, supplements intended for weight loss, as well as use cannabinoid, tobacco, or nicotine products were also excluded from this study. Those who reported any allergies or sensitivities to the product ingredients did not participate in the study.

Participants who qualified were reimbursed for each session they completed at the end of the study. If they did not complete the study, they were reimbursed at a pro-rata rate.

**FIGURE 1:**
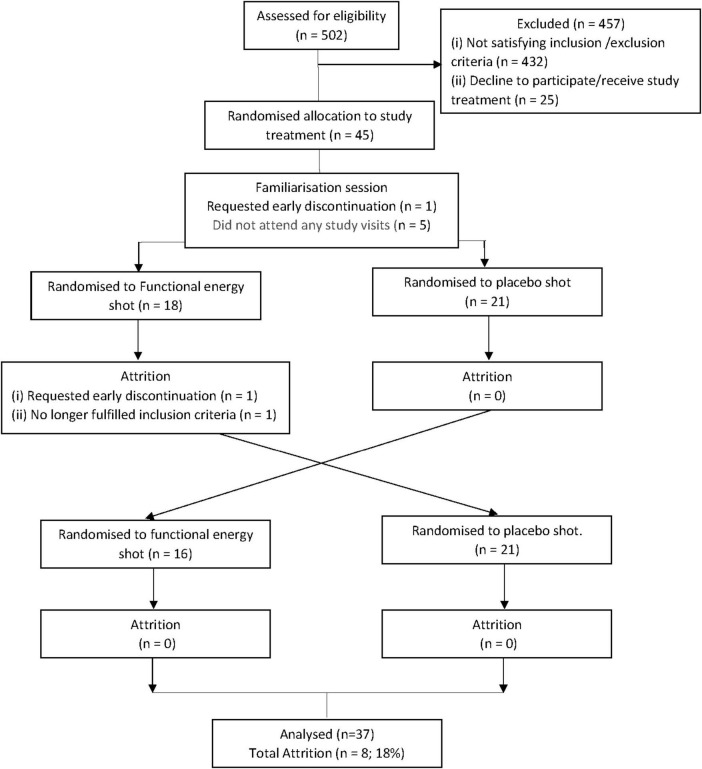
Flow diagram of all participants throughout the study stages.

Of the 45 participants recruited, a total of 37 participants completed all test sessions [13 females, 24 males; mean age: 26.03 (± 3.22) years (18–30 years)]. Five participants did not attend any study visit, one participant attended the familiarization session but requested early discontinuation and two participants attended the familiarization session and first session but one requested early discontinuation and the other was removed as they no longer fulfilled the inclusion criteria. The participants disposition diagram is shown in Figure 1.

This study was conducted according to the principles established in the Declaration of Helsinki. Written informed consent was obtained from all participants in line with the general data protection regulation (GDPR) 2016/679. This study received a full board review and approval by the independent ethical committee of the Sterling IRB, USA (IRB ID: 10423) and the trial was registered at clinicaltrials.gov (NCT06384586).

### Study product

Both the participants and study assessors at site were blind to study product allocation throughout the duration of the trial. All study products were received at site and administered to participants in amber glass jars which were coded by the manufacturer prior to delivery at the site. Participants received each of two products in randomized and counterbalanced order (via a computer-generated pseudo-random permutation procedure in SAS version 9.4.) on separate occasions separated by at least 7 days. The study products were 60 ml shots, each containing four calories, matched for color and flavor, which contained the following:

1. Functional energy shot (*Ryde: Energize*, *The Water Street Collective*, UK): a proprietary blend containing caffeine, taurine, Panax ginseng extract, vitamin B complex, and vitamin C in “tropical” flavor vehicle.
2. Placebo shot: the “tropical” flavor vehicle.

All participants that took part consumed all of the designated study product in its entirety in each session.

### Study outcomes

#### Cognitive Demand Battery

The outcomes making up the CDB correspond closely with the task requirements for measuring the construct of “mental energy” (20). The objective of this battery is to assess the impact of treatment on the speed and accuracy of performance and changes in mood states during continuous performance of cognitively demanding tasks. The battery comprises the performance of a ∼10 min battery of three tasks [Rapid Visual Information Processing (RVIP), Serial 3s, Serial 7s, plus visual analog scales (VAS) measuring aspects of mood states], which are repeated six times in immediate succession (i.e., for a continuous period of 60 min without rest breaks). Application of this battery reliably engenders negative shifts in mood state, including increased mental fatigue, and has been shown to be sensitive to a number of nutritional interventions, including ginseng (12, 13) and water-soluble (B and C) vitamins (21). The tasks making up the CDB are:

*Serial 3s subtraction task* (2 min): computerized 2-min versions of the working memory/executive function serial subtraction tasks. Participants are required to count backwards in threes from a random starting number between 800 and 999 as quickly and as accurately as possible using the touch screen keyboard; after the first subtraction the resulting number is no longer displayed on the screen. The task is scored for number of correct responses and number of errors. In the case of incorrect responses subsequent responses are scored as positive if they were scored as correct in relation to the new number.

*Serial 7s subtraction task* (2 min): this is identical to the serial threes task with the exception that it involves the serial subtraction of sevens.

*Rapid Visual Information Processing task* (5 min): in this classic test of focused attention, the participant monitors a continuous series of digits presented at the rate of 100 per minute for targets of three consecutive odd or three consecutive even digits. The participant responds to the detection of a target string by pressing the response button as quickly as possible. Eight correct target strings are presented in each minute. The task is scored for percentage of target strings correctly detected, average reaction time for correct detections, and number of false alarms.

V*isual Analog Scales (CDB VAS)*: participants complete a series of VAS after each repetition of the CDB tasks. For each VAS they rate how they felt by clicking on a line which is anchored by antonymic mood descriptors. The VAS scales were anchored by: “not at all sociable – extremely sociable”; “not at all mentally fatigued – extremely mentally fatigued”; “not at all alert – extremely alert”; “not at all physically energized – extremely physically energized”; “not at all jittery – extremely jittery”; “unable to relax – extremely relaxed”; and “under-stimulated – over-stimulated.”

#### Additional “working memory” cognitive outcomes

“*Sternberg*” *Numeric Working Memory task*: a series of five single digits to be memorized are displayed on the screen, one at a time. Once the series is complete, 30 single digits are displayed one at a time and participants respond to indicate if each number was presented in the previous list or not. The task is repeated three times with different digits. The task outcomes are % accuracy and reaction time (msec).

“*Corsi Blocks*”: Spatial Working Memory task. Nine blue squares on a black background are displayed on the screen. Some of the blue squares change to red and back to blue again in a sequence. Participants are required to remember this sequence and repeat it by clicking on the squares in the correct order. The task is repeated five times at each level of difficulty with the sequence span increasing from four upward, until the participant can no longer correctly recall the sequences. The task outcome is “span score” and this is calculated as the average of the last three correctly completed trials.

#### Additional mood measures

*Profile of Mood States (POMS)* short form (23): The POMS is a 35-item inventory, the individual item scores of which are collapsed into seven dimensions of mood: anger/hostility, confusion/bewilderment, depression/dejection, fatigue/inertia, tension/anxiety, friendliness and vigor/activity. The POMS also returns a global composite “Total Mood Disturbance” score. Individual items are not analyzed.

The *Self-Assessment Manikin* (SAM) and *Check-all-that-apply* (CATA) were also completed but the data is not reported for brevity.

Participants completed each session on Microsoft Surface Pro 7+ tablets. All cognitive tasks and VAS were completed using the touch-screen version of the Computerized Mental Performance Assessment System (COMPASS version 5.0 – Northumbria University, Newcastle upon Tyne, UK). All remaining questions were completed via a separate online survey link.

### Procedures

A familiarization session was conducted during which participants were screened and given an informed consent form to sign. Participants were then briefed on the requirements of the study and given training and practice on all aspects of the protocol, including three repetitions of the tasks.

Prior to the two subsequent active study sessions, participants consumed a normal-sized meal at least 1 h prior to the start of the study session and then fasted from food and beverage consumption (except water), for 1 h prior to and during the session. Additionally, they abstained from alcohol, caffeine, and exercise starting from the night before each session through to the end of the research session. Participants were not allowed access to their mobile phones during their testing visits.

At the start of each active study session, participants completed a baseline assessment comprising completion of the mood scales and working memory tasks, followed by three completions of the CDB tasks and a single completion of the CDB VAS. Following this, participants consumed the designated study product for that day.

Immediately after consumption of the study product, participants completed several self-report sensorial measures on the product flavor (data not reported here), followed by a 30-min break to allow for ingredient absorption period during which participants watched a travel documentary as a non-stimulating activity.

**FIGURE 2:**
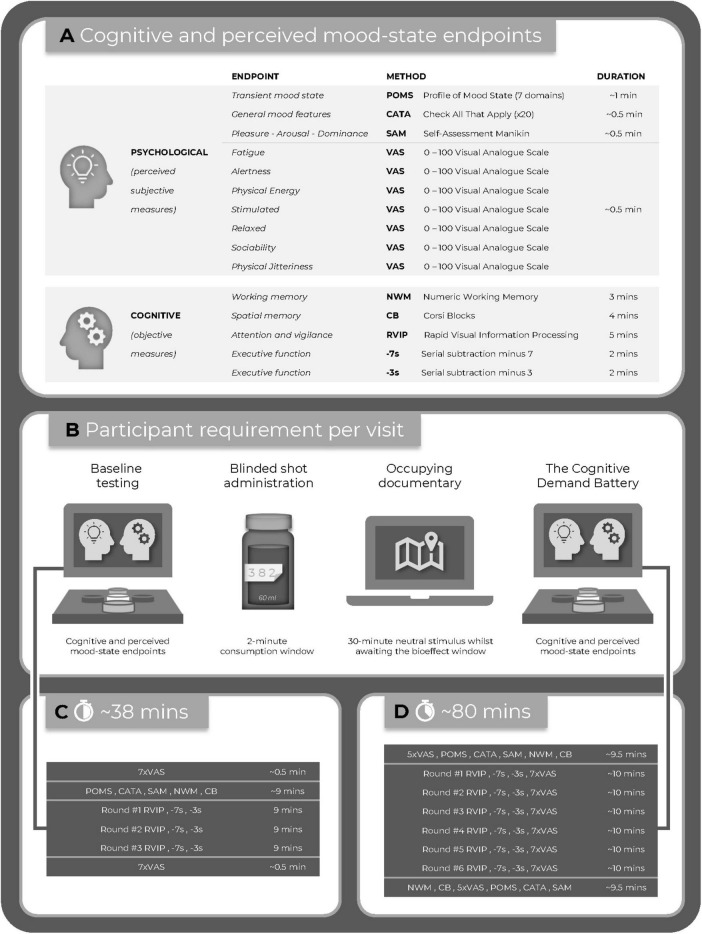
**(A)** The study endpoints; **(B)** the timeline of the active assessment visits; **(C)** the baseline assessment; and **(D)** the post-dose assessment.

Commencing at 30-min post-dose participants completed the mood scales and working memory tasks, followed by six repetitions of the CDB tasks and VAS. Finally, participants completed the mood scales and working memory tasks. The assessment visit methodology is summarized in Figure 2.

### Sample size

A power analysis was conducted using G*Power for sample size estimation, based on data from a prior internal pilot study with the same methodology (*n* = 55), which compared placebo to the prototype product for CDB accuracy. The effect size in this study was 0.61. With a significance criterion of α = 0.05 and power = 0.90, the minimum sample size needed with this effect size was deemed sufficient for Linear Mixed Models (LMM) analysis at *n* = 31. Thus, the target sample size of *n* = 45 was more than adequate to test the study hypothesis, accounting for potential drop-out of participants.

### Analysis

#### Primary and secondary outcomes

The primary outcome for this study was the measurement of “mental energy” via CDB Performance Index for the functional energy shot versus placebo. The secondary outcomes were the measurements of CDB accuracy, the individual assessments of cognitive tasks as well as the mood VAS ratings during the CDB for the functional energy shot versus placebo. All other outcomes were tertiary and included due to the exploratory nature of this this study.

The two composite measures were calculated prior to analysis: CDB Performance Index. This comprised averaged *Z* scores for the principal performance measure for each of the three tasks: RVIP % accuracy, Serial 3s total number correct, and Serial 7s total number correct.

CDB accuracy: this comprised averaged *Z* scores for the principal measures of accuracy of performance for the three tasks: RVIP % accuracy; Serial 3s % errors; and Serial 7s % errors (the latter two were inverted so that a higher *Z* score related to better accuracy).

#### Definition of analysis populations

Given the aims of the study, the appropriate analysis population for the study was decided *a priori* to be the *per protocol* population, consisting of all of the allowable data (after blind-data review) from all of the participants that completed both treatment arms, and who did not violate the inclusion/exclusion criteria.

#### Statistics

The baseline data for the CDB tasks comprised the averaged scores for the three baseline completions of the tasks. The baseline data for the CDB VAS comprised data from the single completion of the scales following the baseline CDB tasks.

All outcomes were analyzed using SPSS (version 24.0, IBM corp.). Prior to the primary analysis of the effects of treatment, pre-dose baseline differences between treatments were investigated by one-way (treatment group) ANOVAS.

For all cognitive and mood measures, the primary analysis of post-dose data was by LMM using the MIXED procedure, using ML estimation in SPSS (version 24.0, IBM corp.) with the pre-dose baseline data for each outcome included as a covariate. For all LMM analyses the covariance structure (from CS, AR1, AD1, and UN) that provided the best fit was evaluated with reference to Schwarz’s Bayesian information criterion (BIC). For the CDB composite scores, task outcomes and VAS scores, terms were fitted for product (functional energy shot, placebo shot) and repetition (6) and their interaction. For the POMS and working memory task outcomes the repetition factor was replaced with post-dose “assessment” factor (pre-CDB, post-CDB).

In the event of a significant main effect or product X assessment interaction effect, a single set of *a priori* planned comparisons (*t*-tests using the pooled variance from the LMM analysis) were undertaken. In order to establish the time-course of any effects, the planned comparisons were conducted on data for each intervention during each repetition or assessment.

Given the experimental aims it was not deemed necessary to adjust these *a priori* comparisons for multiplicity. However, the planned comparisons between intervention means are only reported below for those outcomes that achieved a significant main effect of intervention, or an intervention X repetition/assessment interaction effect.

## Results

### Baseline differences

The only baseline differences in the data collected prior to consuming the study products were seen on the POMS. Participants scored higher on the “depression/dejection” [*F*(1,36) = 5.68, *p* = 0.023] and “tension/anxiety” [*F*(1,36) = 6, *p* = 0.019] domains, and as a consequence of this also on the “total mood disturbance” factor [*F*(1,36) = 5.3, *p* = 0.027] prior to consuming placebo. However, it should be noted that the scores for both groups on all of these measures were very low and comparable to the scores reported in post dose data. There were no other baseline differences on any measure.

### Cognitive Demand Battery

#### Global performance measures

**FIGURE 3:**
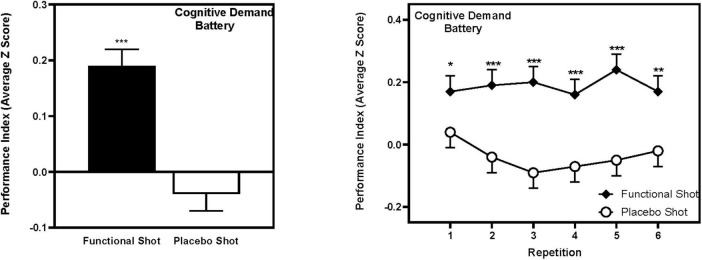
The effects of the functional energy shot and placebo shot on the global CDB Performance Index score (comprising averaged *Z*-scores for RVIP accuracy, Serial 3s correct, and Serial 7s correct). The left-hand panels show the main effect of treatment averaged across the six task repetitions and the right-hand panel shows time course data from each post-dose repetition of the tasks. Data are estimated means from the LMM analysis. Asterisks represent significant planned comparisons between the functional energy shot and the placebo shot at that time point. *p < 0.05; **p < 0.01; ***p < 0.001.

Participants performed significantly better following the functional energy shot, in comparison to the placebo shot, across the CDB tasks as evidenced by the Performance Index score (comprising performance *Z* scores for each task) [*F*(1,74) = 39.3, *p* < 0.001], which was the primary outcome measure. Reference to the planned comparisons of data from each repetition showed that the functional energy shot outperformed placebo at each time point (all *p* < 0.001, except repetition 1, *p* < 0.05, and repetition 6, *p* < 0.01). The CDB Performance Index data is represented graphically in Figure 3.

A similar pattern was also seen for the CDB Accuracy score (comprising accuracy *Z*-scores from each task) [*F*(1,85) = 8.6, *p* = 0.004], although reference to the individual task data suggest this was likely due to both the Performance Index and accuracy measures incorporating RVIP accuracy. The data from both global CDB measures, including comparisons at each repetition are presented in Table 1.

**TABLE 1:** The effects of the functional energy shot and the placebo shot on the Cognitive Demand Battery outcomes.

Outcome		Pre-dose		Post-dose data		Repetition of tasks														
		**Baseline**		**(Averaged = main effect)**		**1**		**2**		**3**		**4**		**5**		**6**				
		**Mean**	**SEM**	**Mean**	**SEM**	**Mean**	**SEM**	**Mean**	**SEM**	**Mean**	**SEM**	**Mean**	**SEM**	**Mean**	**SEM**	**Mean**	**SEM**		***F***	***P***
**Global CDB measures**																				
**CDB** performance (Z)	Functional	−0.14	0.13	0.19	0.03***	0.17	0.05*	0.19	0.05***	0.20	0.05***	0.16	0.05***	0.24	0.05***	0.17	0.05**	shot	39.32	0.000
	Placebo	−0.17	0.13	−0.04	0.03	0.04	0.05	−0.04	0.05	−0.09	0.05	−0.07	0.05	−0.05	0.05	−0.02	0.05	shot*rep	1.38	0.231
**CDB** accuracy (Z)	Functional	0.00	0.10	0.08	0.05**	0.11	0.08	0.20	0.08*	0.15	0.08**	0.06	0.09	0.04	0.08*	−0.05	0.08	shot	8.56	0.004
	Placebo	0.01	0.10	−0.08	0.05	0.05	0.08	−0.03	0.08	−0.14	0.08	−0.08	0.08	−0.18	0.08	−0.12	0.08	shot*rep	1.02	0.408
**Individual task outcomes**																				
**Serial 3s** no. correct	Functional	34.64	2.32	39.63	0.78**	37.65	1.18	39.22	1.18^t^	39.25	1.18^t^	40.60	1.19	40.60	1.18	40.49	1.18	shot	7.65	0.007
	Placebo	33.07	2.32	37.58	0.79	36.58	1.18	36.72	1.18	36.75	1.18	38.42	1.18	38.81	1.19	38.18	1.19	shot*rep	0.17	0.972
**Serial 3s** errors	Functional	2.70	0.24	3.31	0.27	3.29	0.45	2.51	0.45	3.40	0.45	3.00	0.46	3.89	0.45	3.78	0.45	shot	0.13	0.72
	Placebo	2.63	0.24	3.41	0.27	2.88	0.45	3.53	0.45	3.48	0.45	3.15	0.45	3.95	0.46	3.49	0.46	shot*rep	0.90	0.48
**Serial 7s** no. correct	Functional	20.06	1.56	24.39	0.43**	23.95	0.76	23.68	0.76	24.95	0.76**	23.73	0.77	25.49	0.76*	24.55	0.76	shot	7.75	0.006
	Placebo	19.28	1.56	22.86	0.43	22.81	0.76	22.76	0.76	22.11	0.78	22.65	0.76	23.05	0.77	23.77	0.77	shot*rep	1.10	0.361
**Serial 7s** errors	Functional	2.36	0.23	2.69	0.23	2.66	0.37	2.36	0.37	2.47	0.37	2.81	0.37	2.69	0.37	3.15	0.37	shot	0.19	0.67
	Placebo	2.32	0.23	2.60	0.23	2.44	0.37	2.09	0.37	2.93	0.38	2.55	0.37	2.74	0.37	2.85	0.37	shot*rep	0.49	0.78
**RVIP** % correct	Functional	49.26	3.79	56.41	1.45***	58.87	1.96*	58.60	1.96***	56.98	1.96***	54.34	1.96***	56.03	1.96***	53.66	1.96***	shot	58.55	0.000
	Placebo	50.92	3.79	46.27	1.45	53.39	1.96	47.64	1.96	46.16	1.96	42.64	1.96	43.39	1.96	44.41	1.98	shot*rep	1.60	0.160
**RVIP** speed (msec)	Functional	556.6	8.19	542.8	5.3***	538.9	7.7	545.6	7.7	549.7	7.7^t^	543.6	7.7	539.8	7.7^t^	538.9	7.7**	shot	13.5	0.000
	Placebo	552.0	8.19	559.3	5.3	551.2	7.7	560.4	7.7	567.3	7.7	556.1	7.7	557.3	7.7	563.7	7.7	shot*rep	0.3	0.936
**RVIP** false alarm	Functional	6.60	1.45	6.94	0.90	6.39	1.04	6.58	1.04	6.31	1.04	7.36	1.04	6.80	1.04	8.23	1.04	shot	0.66	0.42
	Placebo	7.59	1.45	6.60	0.90	5.64	1.04	7.29	1.04	6.75	1.04	6.78	1.04	6.97	1.04	6.14	1.05	shot*rep	1.75	0.12

The baseline data is the average score across the three pre-dose repetitions of the tasks. Baseline data are raw scores (+SEM) and post-dose data are estimated means (+SEM) from the LMM analysis. The gray column presents data averaged across the post-dose repetitions, therefore corresponding to the main effect of treatment. Asterisks denote a significant difference to placebo on the planned comparison at that time point: *p < 0.05; **p < 0.01; ***p < 0.001; *^t^p* < 0.1. The right-hand columns contain the F scores and probabilities relating to the LMM analysis main effect of intervention (shot) and shot*repetition interaction effects.

#### Individual tasks

In keeping with the global improvement seen in terms of the CDB Performance Index, following the functional energy shot participants performed significantly better than following the placebo shot across repetitions on all three CDB tasks; RVIP accuracy [*F*(1,78) = 58.5, *p* < 0.001], RVIP speed [*F*(1,91) = 13.5, *p* < 0.001], Serial 3s number correct [*F*(1,81) = 7.6, *p* = 0.007], and Serial 7s number correct [*F*(1,94) = 7.7, *p* = 0.006].

**FIGURE 4:**
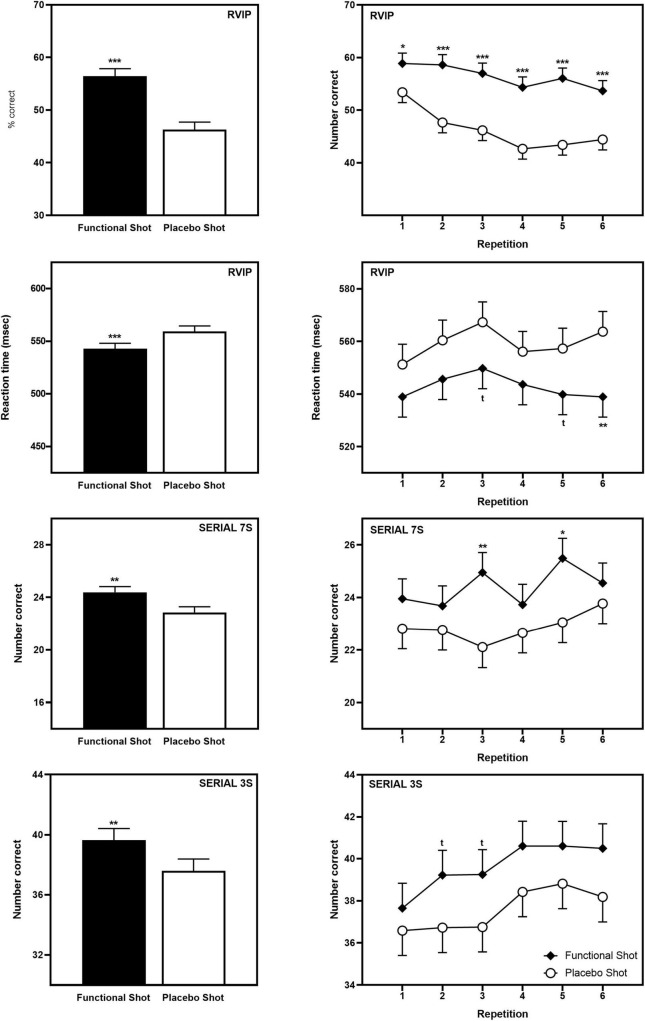
The effects of the functional energy shot and placebo shot on performance of the Individual CDB tasks (RVIP accuracy, RVIP speed, Serial 3s correct, and Serial 7s correct). The left-hand panels show the main effect of treatment averaged across the six task repetitions and the right-hand panel shows time course data from each post-dose repetition of the tasks. Data are estimated means from the LMM analysis. Asterisks represent significant planned comparisons between the functional energy shot and the placebo shot at that time point. *p < 0.05; **p < 0.01; ***p < 0.001; *^t^p* < 0.1.

Reference to the planned comparisons of data from each repetition showed that the functional energy shot outperformed placebo at all time points in terms of RVIP accuracy (all repetitions *p* < 0.001 except first, *p* < 0.05), with isolated significant and trend-level differences on the other tasks (see Figure 4). The data for the CDB outcomes that evinced significant differences is presented graphically in Figure 4 and the data for all CDB outcomes is presented in Table 1.

#### Mood visual analog scale ratings during the CDB

Consuming the functional energy shot significantly attenuated the negative consequences of extended performance of the cognitively demanding tasks that were seen in the placebo condition. The effect was seen across repetitions following the functional energy shot in terms of significantly reduced mental fatigue [*F*(1,88.8) = 12.7, *p* < 0.001], increased alertness [*F*(1,67.1) = 23.3, *p* < 0.001], increased feelings of being physically energized [*F*(1,77) = 13.8, *p* < 0.001], and increased ratings of being “sociable” [*F*(1,73.5) = 6.7, *p* = 0.012] in comparison to placebo. Reference to the planned comparisons at each repetition showed that this effect reached significance during several repetitions for mental fatigue (first/sixth – *p* < 0.05, third – *p* < 0.01), and physically energized (third/fourth/fifth – *p* < 0.01, sixth – *p* < 0.05) and during all repetitions for alertness (first/sixth – *p* < 0.05, second – *p* < 0.001, third/fourth/fifth – *p* < 0.01).

Although neither measure exhibited a pattern suggesting they were being modulated by extended task performance, across repetitions the functional energy shot also resulted in increased ratings of “stimulated” [*F*(1,58) = 4.1, *p* = 0.47] and also increased ratings of “jittery” [*F*(1,69.5) = 25.6, *p* < 0.001] in comparison to placebo. The findings with regards “jittery” were also evident during each repetition of the tasks (first – *p* < 0.05, second/fifth/sixth – *p* < 0.01, third/fourth – *p* < 0.001). Note also that the ratings of “jittery” following the functional energy shot may be significantly greater than placebo, but they were also still entirely in the “not at all jittery” half of the scale.

**FIGURE 5:**
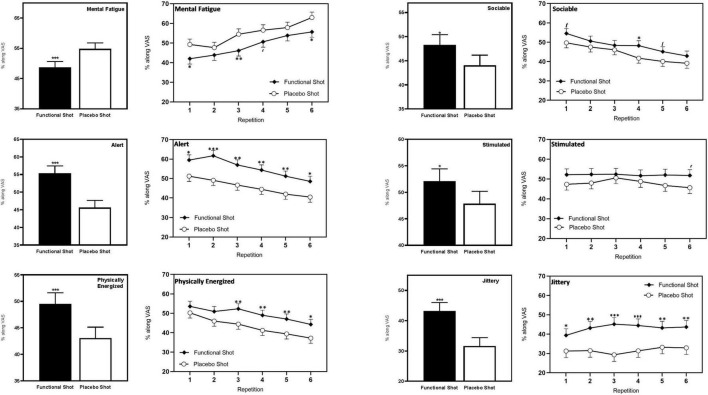
The effects of the functional energy shot and placebo shot on VAS ratings of mood state during the CDB. For each outcome the left-hand panels show the main effect of treatment averaged across the six task repetitions and the right-hand panel shows time course data from each post-dose repetition of the tasks. Data are estimated means from the LMM analysis. Asterisks represent significant planned comparisons between the functional energy shot and the placebo shot at that time point. *p < 0.05; **p < 0.01; ***p < 0.001.

The data from the VAS that evinced a significant effect of the shots are shown in Figure 5. Data for all of the VAS, including the results of planned comparisons are presented in Table 2.

**TABLE 2:** The effects of the functional energy shot and the placebo shot on mood VAS during the Cognitive Demand Battery.

Outcome		Pre-dose		Post-dose data		Repetition of tasks														
		Baseline		(Averaged = main effect)		1		2		3		4		5		6			*F*	*P*
		Mean	SEM	Mean	SEM	Mean	SEM	Mean	SEM	Mean	SEM	Mean	SEM	Mean	SEM	Mean	SEM			
**Mental fatigue**	Functional	51.46	3.58	48.68	1.99***	41.99	2.74*	43.85	2.74	46.12	2.74**	50.64	2.74^t^	53.80	2.74	55.67	2.74*	shot	12.67	0.001
	Placebo	55.68	3.58	54.80	1.99	49.27	2.74	47.65	2.74	54.49	2.74	56.54	2.74	57.87	2.74	63.00	2.76	shot*rep	0.62	0.688
**Alertness**	Functional	47.11	3.72	55.35	2.06***	59.48	2.68*	61.69	2.68***	56.94	2.68**	54.32	2.68**	51.18	2.68**	48.48	2.68*	shot	23.27	0.000
	Placebo	44.73	3.72	45.60	2.06	51.17	2.68	49.04	2.68	46.60	2.68	44.42	2.68	41.93	2.68	40.42	2.70	shot*rep	0.55	0.741
**Physically energized**	Functional	41.73	3.31	49.51	2.08***	53.54	2.63	50.92	2.63	52.30	2.63**	48.97	2.63**	47.05	2.63**	44.27	2.63*	shot	13.83	0.000
	Placebo	37.68	3.31	43.05	2.08	50.20	2.63	45.96	2.63	44.39	2.63	41.18	2.63	39.36	2.63	37.19	2.65	shot*rep	0.37	0.870
**Stimulated**	Functional	42.38	3.38	52.07	2.35*	52.18	2.93	52.31	2.93	52.42	2.93	51.67	2.93	52.04	2.93	51.80	2.93^t^	shot	4.10	0.047
	Placebo	44.16	3.38	47.83	2.35	47.35	2.93	47.95	2.93	50.60	2.93	48.76	2.93	46.71	2.93	45.64	2.95	shot*rep	0.32	0.904
**Relaxed**	Functional	42.24	2.85	42.37	2.05	47.70	2.70	43.91	2.70	41.81	2.70	41.46	2.70	40.43	2.70	38.91	2.70	shot	1.01	0.319
	Placebo	44.30	2.85	44.55	2.05	48.33	2.70	48.19	2.70	44.55	2.70	42.71	2.70	41.68	2.70	41.86	2.72	shot*rep	0.40	0.849
**Sociable**	Functional	46.30	3.40	48.29	2.13*	54.55	2.57^t^	50.60	2.57	48.36	2.57	48.23	2.57*	45.15	2.57^t^	42.88	2.57	shot	6.69	0.012
	Placebo	39.97	3.40	44.03	2.13	49.69	2.57	47.52	2.57	46.09	2.57	41.74	2.57	40.06	2.57	39.10	2.59	shot*rep	0.64	0.667
**Jittery**	Functional	26.32	3.56	43.17	2.82***	39.37	3.40*	43.16	3.40**	45.16	3.40***	44.45	3.40***	43.24	3.40**	43.67	3.40**	shot	25.62	0.000
	Placebo	25.11	3.56	31.58	2.82	31.24	3.40	31.45	3.40	29.32	3.40	31.35	3.40	33.21	3.40	32.91	3.42	shot*rep	0.85	0.515

The baseline data is the score obtained at the end of the baseline CDB tasks. Baseline data are raw scores (+SEM) and post-dose data are estimated means (+SEM) from the LMM analysis. The gray column presents data averaged across the post-dose repetitions, therefore corresponding to the main effect of treatment. Asterisks denote a significant difference to placebo on the planned comparison at that time point: *p < 0.05; **p < 0.01; ***p < 0.001; *^t^p* < 0.1. The right-hand columns contain the F scores and probabilities relating to the LMM analysis main effect of intervention (shot) and shot*repetition interaction effects.

#### Working memory outcomes

**FIGURE 6:**
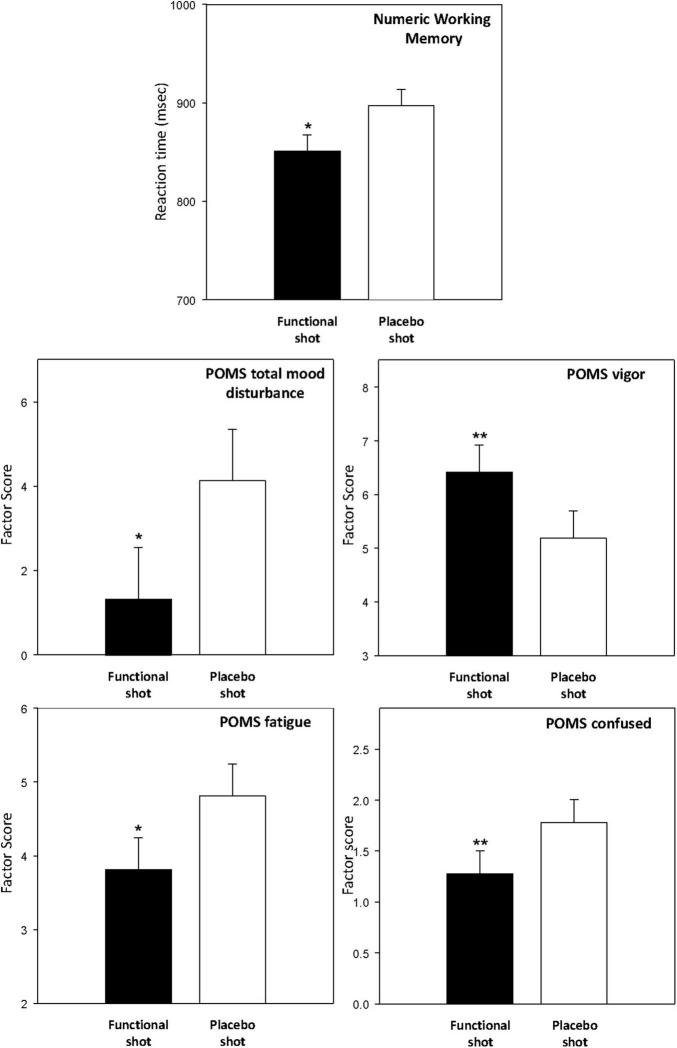
The effects of the functional energy shot and placebo shot on the Numeric Working Memory task (top) and the POMS factors. For each outcome the graph shows the main effect of treatment averaged across the two post-dose assessments. Data are estimated means from the LMM analysis. Asterisks represent significant planned comparisons between the functional energy shot and the placebo shot. *p < 0.05; **p < 0.01; ***p < 0.001; *^t^p* < 0.1.

The two working memory tasks (*Corsi blocks* and *Numeric Working Memory* task) were completed at baseline and both prior to and after the CDB. The functional energy shot resulted in significantly faster performance of the Numeric Working Memory task [*F*(1,139) = 5.6, *p* = 0.02] across the post-dose assessments. This finding is represented graphically in Figure 6. The data from the working memory tasks is presented in Table 3.

**TABLE 3:** The effects of the functional energy shot and the placebo shot on the working memory tasks.

Outcome		Pre-dose		Post-dose data								
		Baseline		(Averaged = main effect)		Pre- CDB		Post-CDB			*F*	*P*
		Mean	SEM	Mean	SEM	Mean	SEM	Mean	SEM			
**Corsi blocks span score**	Functional	6.31	0.16	6.15	0.14	6.08	0.17	6.21	0.17	shot	0.29	0.59
	Placebo	6.19	0.16	6.07	0.14	6.18	0.17	5.96	0.17	shot*ass	1.45	0.23
**NWM % accuracy**	Functional	96.76	0.85	96.36	0.74	96.73	0.86	95.98	0.86	shot	0.20	0.65
	Placebo	95.86	0.85	96.07	0.74	96.12	0.86	96.02	0.87	shot*ass	0.28	0.60
**NWM speed (msec)**	Functional	906.8	34.0	850.9	16.3*	865.8	19.5^t^	836.0	19.5^t^	shot	5.6	0.02
	Placebo	914.2	34.0	897.1	16.4	910.4	19.5	883.7	19.7	shot*ass	0.0	0.92

Baseline data are raw scores (+SEM) and post-dose data are estimated means (+SEM) from the LMM analysis. The gray column presents data averaged across the post-dose repetitions, therefore corresponding to the main effect of treatment. Asterisks denote a significant difference to placebo on the planned comparison at that time point: *p < 0.05; ^t^*p* < 0.1. The right-hand columns contain the F scores and probabilities relating to the LMM analysis main effect of intervention (shot) and shot*assessment interaction effects.

#### Profile of Mood States

The POMS was completed at baseline and both prior to the working memory tasks and after the CDB. The functional energy shot was associated with improvements to several of the mood factors derived from the POMS across the two post-dose assessments in comparison to the placebo shot. These benefits were seen in terms of increased vigor/activity [*F*(1,43.8) = 7.6, *p* = 0.009], decreased fatigue [*F*(1,49.3) = 5, *p* = 0.03], decreased “anger/hostility” [*F*(1,59.9) = 5.6, *p* = 0.02], decreased confusion/bewilderment [*F* (1,42.7) = 8.1, *p* = 0.007], and decreased “total mood disturbance” [*F*(1,49) = 6.7, *p* = 0.013]. The averaged data for these outcomes are presented graphically in Figure 6. The data for all of the POMS factors, including the results of planned comparisons of data from both post-dose assessments are presented in Table 4.

**TABLE 4:** The effects of the functional energy shot and the placebo shot on the profile of Mood States (POMS) outcomes.

Outcome		Pre-dose		Post-dose data								
		Baseline		(Averaged = main effect)		Pre- CDB		Post-CDB			*F*	*P*
		Mean	SEM	Mean	SEM	Mean	SEM	Mean	SEM			
**Total mood disturbance**	Functional	−3.14	1.49	1.32	1.22*	−1.82	1.55	4.47	1.55*	shot	6.71	0.013
	Placebo	0.05	1.49	4.13	1.22	0.29	1.55	7.97	1.56	shot*ass	0.33	0.57
**Friendliness**	Functional	10.62	0.79	9.38	0.37	10.01	0.44	8.74	0.44	shot	2.75	0.10
	Placebo	10.59	0.79	8.75	0.38	9.28	0.44	8.22	0.45	shot*ass	0.09	0.77
**Vigor / Activity**	Functional	7.41	0.73	6.42	0.50**	7.12	0.57^t^	5.71	0.57*	shot	7.57	0.009
	Placebo	6.78	0.73	5.19	0.51	5.94	0.57	4.44	0.58	shot*ass	0.01	0.91
**Fatigue / Inertia**	Functional	1.73	0.49	3.81	0.43*	2.58	0.57	5.04	0.57*	shot	5.04	0.029
	Placebo	2.81	0.49	4.81	0.44	3.24	0.57	6.38	0.57	shot*ass	0.50	0.48
**Depression / Dejection**	Functional	0.14	0.14	0.42	0.11	0.26	0.13	0.59	0.13	shot	0.35	0.56
	Placebo	0.46	0.14	0.38	0.11	0.25	0.13	0.50	0.14	shot*ass	0.17	0.68
**Confusion / Bewilderment**	Functional	1.00	0.24	1.28	0.22**	0.93	0.31	1.63	0.31*	shot	8.06	0.007
	Placebo	1.27	0.24	1.78	0.23	1.27	0.31	2.30	0.31	shot*ass	0.52	0.48
**Tension / Anxiety**	Functional	1.08	0.26	1.80	0.27	1.34	0.33	2.26	0.33	shot	2.34	0.13
	Placebo	1.70	0.26	1.41	0.27	1.00	0.33	1.82	0.33	shot*ass	0.04	0.85
**Anger / Hostility**	Functional	0.32	0.19	0.46	0.14*	0.22	0.19	0.70	0.19*	shot	5.62	0.021
	Placebo	0.59	0.19	0.90	0.14	0.44	0.19	1.36	0.20	shot*ass	1.37	0.24

Baseline data are raw scores (+SEM) and post-dose data are estimated means (+SEM) from the LMM analysis. The gray column presents data averaged across the post-dose repetitions, therefore corresponding to the main effect of treatment. Asterisks denote a significant difference to placebo on the planned comparison at that time point: *p < 0.05; **p < 0.01; ^t^*p* < 0.1. The right-hand columns contain the F scores and probabilities relating to the LMM analysis main effect of intervention (shot) and shot*assessment interaction effects.

Data were also collected at the same time as the POMS using the SAM and CATA. For brevity this data is not reported here.

## Discussion

The results of the current study demonstrate that acute administration of a functional energy shot (*Ryde: Energize*) can improve markers of mental energy such as perceived alertness, physical energy and stimulation. Additionally, improvement in cognitive performance including focused attention and working memory were observed while also reducing the impact of perceived mental fatigue during a mentally demanding cognitive task.

Modulation of cognitive performance was observed in terms of both improved accuracy and speed of performance. The results showed that consuming the functional energy shot resulted in a significant improvement over placebo for the primary outcome, CBD Performance Index (a global measure of task performance derived from the CDB task data), and CDB accuracy (a global measure of accuracy of performance and specific improvements on all three tasks that make up the battery). These task specific improvements were seen in both the accuracy and speed of the RVIP task, which measures focused attention, and in the overall performance (i.e., number correct) of both the Serial 7s and Serial 3s tasks. These latter tasks load heavily on both working memory and executive function. It is also noteworthy that there was no loss of accuracy, as assessed by the number of RVIP false alarms and the number of incorrect serial subtractions. In addition to the findings with regards to the CDB, the functional energy shot also improved the performance of one of two working memory tasks that were performed before and after the CDB, with enhanced speed seen on the Numeric Working Memory task. However, it should be noted that there were no significant differences between conditions in Numeric Working Memory task accuracy or performance in visuospatial working memory as assessed using the Corsi blocks task. There was no evident time restriction to the cognitive benefits of the functional energy shot, with significant beneficial effects extending throughout the six repetitions of the tasks.

In terms of the functional energy shot’s effects on mood states, the most striking finding was of an attenuation of the negative effects of extended completion of the CDB. In this respect, in comparison to placebo, the functional energy shot alleviated the steadily increasing mental fatigue, and steadily decreasing ratings of alertness, sociability and “being physically energized” experienced by participants during the extended period of task performance. The functional energy shot also improved ratings of “being stimulated” and increased ratings of jitteriness in comparison to placebo. Neither of these latter measures exhibited a directional trend during the CDB. It is also worth noting that the finding regarding jitteriness could potentially be interpreted as an undesirable effect. However, jitteriness was measured on a VAS anchored by “not at all jittery” and “extremely jittery” and the increase seen on this scale following the functional energy shot still remained firmly in the “not at all jittery” half of the scale.

The functional energy shot also enhanced mood as assessed by the POMS questionnaire, which was completed both before the CDB and at the end of testing. Here mood state benefits, in comparison to placebo, were seen in terms of increased “vigor-activity” and reduced “fatigue,” “confusion-bewilderment,” “anger-hostility,” and “total mood disturbance.” In all cases the functional energy shot’s beneficial effects were most marked (and significant on the planned comparisons) during the post-CDB assessment at the end of testing, suggesting again that the shot attenuated the negative impact of the challenging testing protocol.

These results raise the question of which components of the functional energy shot were responsible for the beneficial effects evinced here. It might be tempting to attribute them to the caffeine content. Whilst caffeine taken by itself does have relatively consistent effects, these are restricted to increased subjective alertness/arousal and, in terms of mental performance, consistent improvements in the performance of tasks assessing reaction time or focused attention/vigilance. Caffeine’s effects do not generally extend to other cognitive domains, and it has inconsistent effects on working memory and executive function tasks, with evidence suggesting that it can impair the performance of more complex tasks (6, 19). In this context caffeine would not be expected to have a marked effect on performance of the CDB tasks, with the potential exception of the RVIP focused attention task. Indeed, several studies have assessed the effects of caffeine alone using the CDB paradigm. These studies reported that psychoactive doses of caffeine (66–75 mg) had no effect on performance of the CDB tasks (24–26), or benefits that were restricted to improved performance of a single task (Serial 7s) (27). Similarly, whilst caffeine has the potential to increase alertness or reduce mental fatigue, caffeine alone is not associated with broader benefits to mood, including when mood is assessed by the POMS (28–30). The magnitude of these benefits seen following the functional energy shot in the current study are therefore not in keeping with the effects of caffeine alone.

This raises the question of which components of the functional energy shot are responsible for the broad range of effects seen here. Ginseng has been shown to have consistent independent beneficial effects across multiple cognitive domains after a single dose (10). These benefits include attenuated mental fatigue and improved performance of the CDB (12, 13). Similarly, water-soluble vitamins (B vitamins and vitamin C) have been shown to modulate brain function after a single dose and engender mood and cognitive benefits following longer-term supplementation (15, 16). Examples include attenuated mental fatigue, improved mood, and enhanced task performance during the CDB and other mentally challenging testing paradigms (21, 31). Interestingly, these three components work via very different mechanisms. In the case of caffeine, its effects are predominantly due to modulation of neurotransmission via the prevention of adenosine’s inhibitory effects on neural activity due to caffeine’s binding to adenosine A_1_ and A_2_ receptors (19). Ginseng, on the other hand, likely owes its beneficial effects to interactions between ginsenosides and mammalian steroidal hormonal systems throughout the body, including the stress-responsive glucocorticoid system. This ubiquitous signaling system has the potential for multifarious interactions with brain function (10). Finally, the water-soluble vitamins are key rate-limiting co-factors in both catabolic and anabolic cellular metabolism, with a particular role in the synthesis of neurotransmitters and the regulation of cerebral blood-flow via, for instance, the synthesis of nitric oxide (15). These very different modes of action may facilitate additive effects of this combination of compounds. However, it should also be noted that caffeine’s primary role here may not be predicated on its own independent effects on neurotransmission, but rather its interactive and synergistic properties when co-consumed with other bioactive compounds. These interactive effects are driven by caffeine’s structural similarity to the ubiquitous energy and signaling molecule adenosine and include the inhibition of multiple enzymes that are involved in neurotransmission, cellular homeostasis and signal propagation, and the modulation of the pharmacokinetics and pharmacodynamics of other endogenous and exogenous bioactive molecules, in part via interactions with shared Cytochrome P450 enzymes (19).

Unfortunately, one limitation of the current study is that it was not conceived methodologically in such a way as to disentangle the contributions of its components to their combined effects. The time course of the post-dose assessment period was also too short at ∼2 h to capture any information on the maximum duration of the functional energy shot’s effects. This was an initial study which focused on the potential effects of the functional energy shot on the “mental energy” construct (20). There is therefore a clear opportunity to expand research into the effects of this functional energy shot to include both other testing paradigms, longer testing periods, and the fractionation of the contribution of the component bioactive ingredients. Similarly, further research based on repeated daily use and into the mechanisms underlying the observed beneficial effects, such as through the use of biomarker and neuroimaging studies, could provide further useful insights.

## Conclusion

In conclusion, the present study demonstrated that a functional energy shot (*Ryde: Energize*) containing a unique and proprietary combination of caffeine, taurine, ginseng, and water-soluble vitamins engendered consistent cognitive and mood state benefits that can be interpreted as reflecting increased “mental energy.” Notably, the functional energy shot demonstrated improvements in accuracy and speed across multiple cognitive domains, mitigated negative effects, and enhanced mood. While the specific contributions of each component remain unclear, the study suggests that the combined effects of this blend of caffeine, ginseng, and water-soluble vitamins may synergistically account for the observed benefits.

## Data Availability

The raw data supporting the conclusions of this article will be made available by the authors, without undue reservation.
